# Rapid growth atypical mycobacteria infection associated with growth hormone injections: a case report

**DOI:** 10.1099/acmi.0.000280

**Published:** 2021-11-08

**Authors:** Christian David Cardozo Lomaquiz, Tamara Frontanilla, Natalia Scavone, Alba Fretes, Nathalia Torales, María Elena Pereira, Herminia Mino de Kaspar, Xavier Ortiz, Renate Henning

**Affiliations:** ^1^​ DermatoCenter Clinic, Ciudad del Este, Paraguay; ^2^​ School of medicine, University of São Paulo, São Paulo, Brazil; ^3^​ Microbiología Clínica - Díaz Gill Medicina Laboratorial, Asunción, Paraguay; ^4^​ Microbiología Clinica- Centro Nacional del Quemado y Cirugías Recostructivas, Asunción, Paraguay; ^5^​ Department of Ophthalmology, Ludwig-Maximilians-University of Munich, Munich, Germany; ^6^​ Director of Díaz Gill Medicina Laboratorial, Asunción, Paraguay

**Keywords:** *Mycobacterium Infections*, *Nontuberculous Mycobacteria*, Hormones, Paraguay

## Abstract

**Introduction:**

Infections caused by fast growing mycobacteria have increased markedly worldwide. They are normally associated with trauma, surgery or cosmetic interventions. Paraguay has a deficit in sanitary control including clinics, private practices, and aesthetic centres. This situation is accompanied by the easy access to drugs, which leads to the performance of exclusively medical aesthetic procedures by people without professional knowledge or training.

**Case report:**

A 26-year-old female patient comes to a medical consultation with pain and bruising in the abdominal area with more than 3 months of progression, without fever or apparent cause. Later, she confessed to the application of subcutaneous injections of ‘growth hormones’ at the gym. Excisional biopsy of the lesions was carried out for anatomopathological and microbiological studies. In addition, the use of polymerase chain reaction analysis was indicated because of the strong suspicion of an atypical mycobacterial infection. The Ziehl-Neelsen staining was negative for BAAR, and the PAS-Hematoxylin negative for fungal elements. When performing the culture, the growth of atypical mycobacteria was observed on chocolate and blood agar medium culture. Through the polymerase chain reaction study, it was possible to identify the atypical mycobacterium as *‘Mycobacterium abscessus’*.

**Conclusion:**

The irresponsible application of medications by people without professional authorization or biosafety precautions can lead to the development atypical infections that are difficult to diagnose and treat. This situation could lead to serious complications and even death.

## Introduction

Rapidly growing mycobacteria (RGM), also called nontuberculous, are common in nature, distributed in water, soil, animals and other environments [1]. The *‘Mycobacterium abscessus complex (MABSC)’*, a type of RGM, is an extremely resistant opportunistic pathogen. It is able to survive in the absence of nutrients and in a wide range of temperatures. It can contaminate water supplies, reagents, and cause all kinds of infections [2].

Worldwide, an increase of mycobacterial infections has been observed [3], probably because of the demand for surgeries and cosmetic procedures, as well as immunosuppressed patients, and advances in diagnostic methods, among others. The *MABSC* are found predominantly in localized infections, like extensive tissue trauma, post-traumatic injuries, surgical procedures, chronic pulmonary infections, etc. [2, 4].

Infections caused by *MABSC* show a lot of clinical presentations, being able to affect any organ or system. However, the symptoms and signs are different depending on the species involved [5].

The *MABSC* are particularly difficult to diagnose because they normally do not grow in regular cultures and also, are resistant to disinfectants and some conventional antibiotics, *MABSC* as well as other RGM can cause serious and even fatal infections in humans [6].

Paraguay has a deficit in the regulation and sanitary control of clinics, private practices, aesthetic centres, etc. [7]. This situation and the easy access to drugs, facilitates the performance of aesthetic procedures by people without the training and knowledge. This situation can trigger a number of complications like infections.

Actually, genotypic identification by molecular biology is the best approach for the differentiation of bacterial species [8]. The importance of identification lies in the treatment. There are specific antibiotics for each family of atypical mycobacteria, because they are resistant to most antibiotic drugs and monotherapy may be inadequate [9].

The incorrect use of antibiotics can increase the possibility to developing bacterial resistance [10]. This difficulty in treating infections caused by these mycobacteria can lead to serious health complications, including death.

The objective of this work is to present a case of cutaneous infection with fast growing atypical mycobacteria associated with injections of growth hormones in an adult female patient.

## Case report

**Fig. 1.**
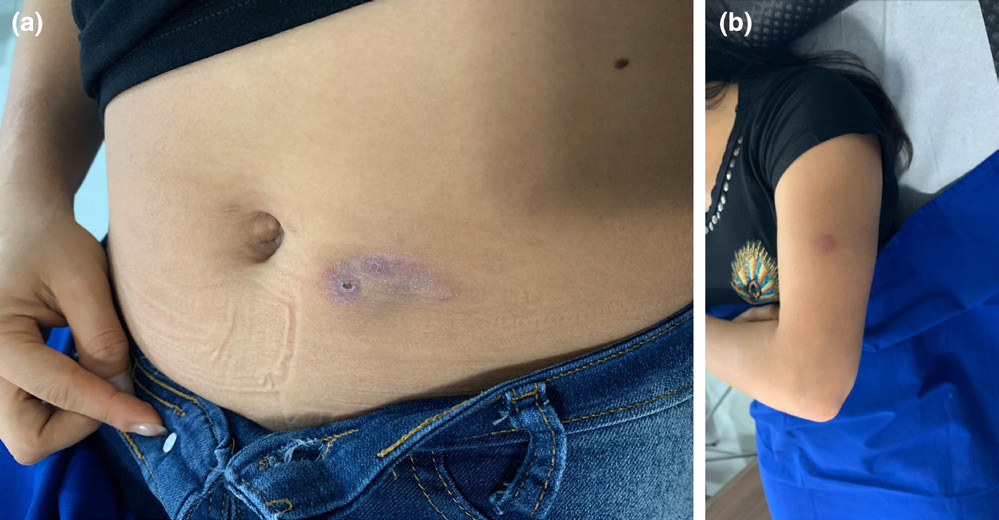
Macroscopic observation of the lesions. Periumbilical region (**a**), left arm (**b**).

This work was approved by the Ethical Committee of National University of Caaguazú. A 26-year-old female patient comes to a medical consultation with pain and bruising in the abdominal area. During the clinical examination, painful, erythematous-violet subcutaneous nodules in the periumbilical region and left arm were found (Fig. 1). No signs and symptoms of lung disease were detected. During palpation, no adenomegaly was found. The patient reported 3 months of evolution, with no fever or apparent cause.

After an insistent interrogation, she confessed the application of subcutaneous injections of ‘growth hormones’ in the gym, months before the symptoms appeared.

Excisional biopsy of the lesions was carried out for anatomopathological and microbiological studies. In addition, polymerase chain reaction (PCR) analysis was indicated because the strong suspicion of an atypical mycobacteriosis.

**Fig. 2.**
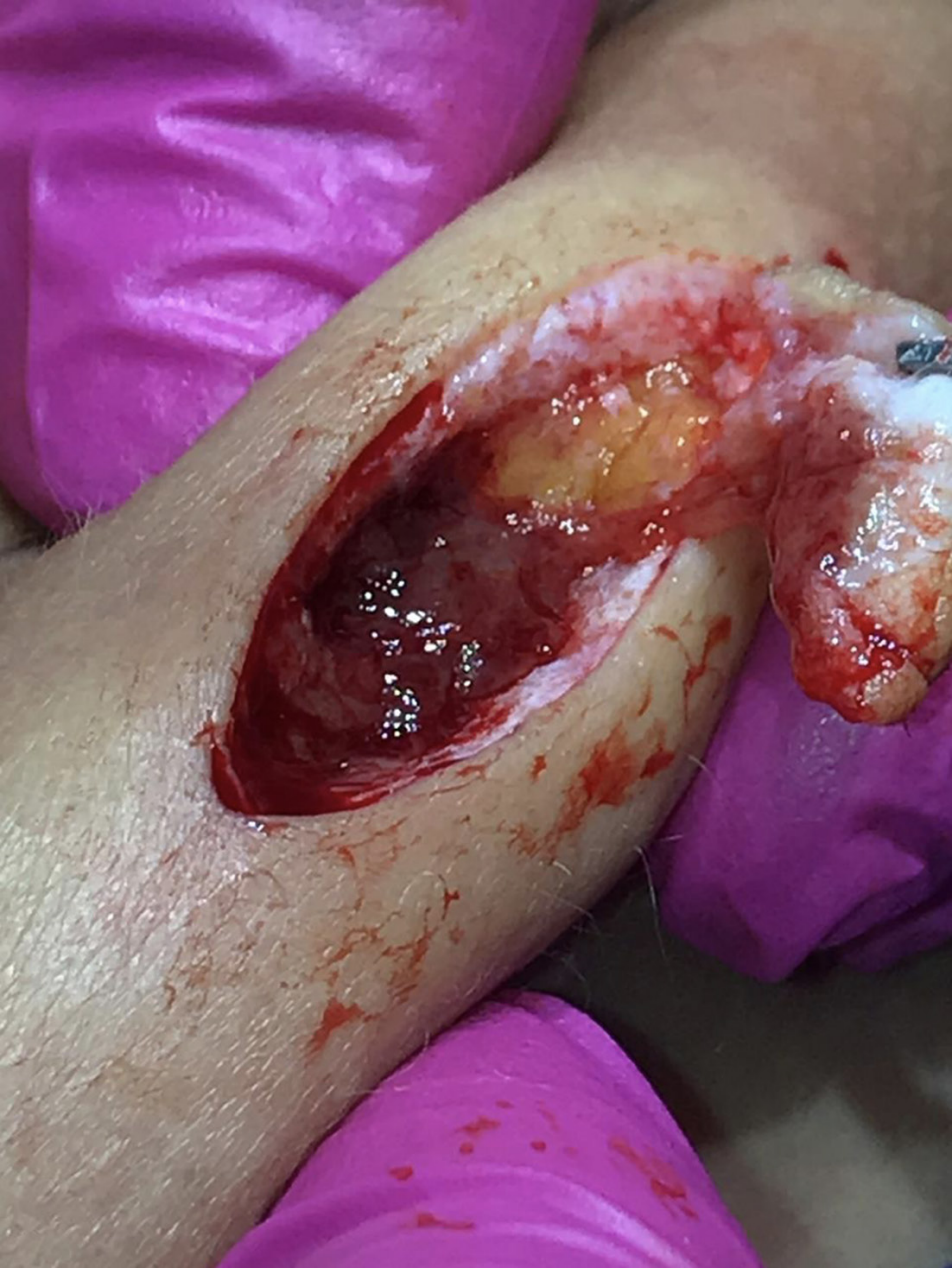
Excision biopsy of the skin lesions.

**Fig. 3.**
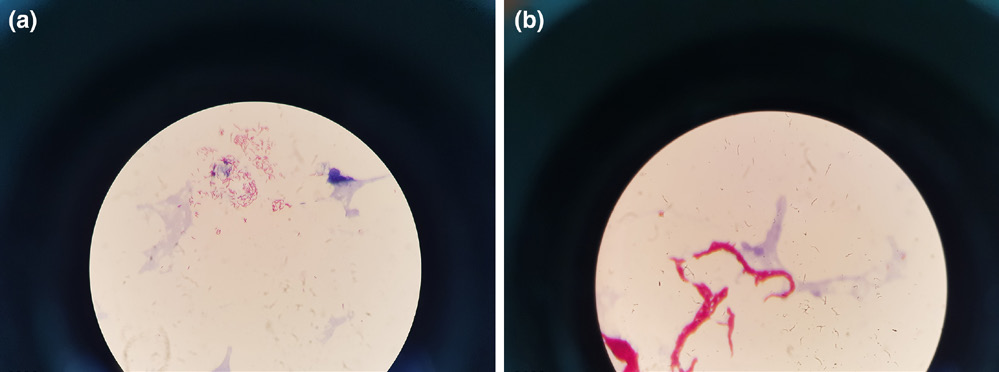
Ziehl-Neelsen staining of the thioglycollate enrichment medium. We observe BAAR.

**Fig. 4.**
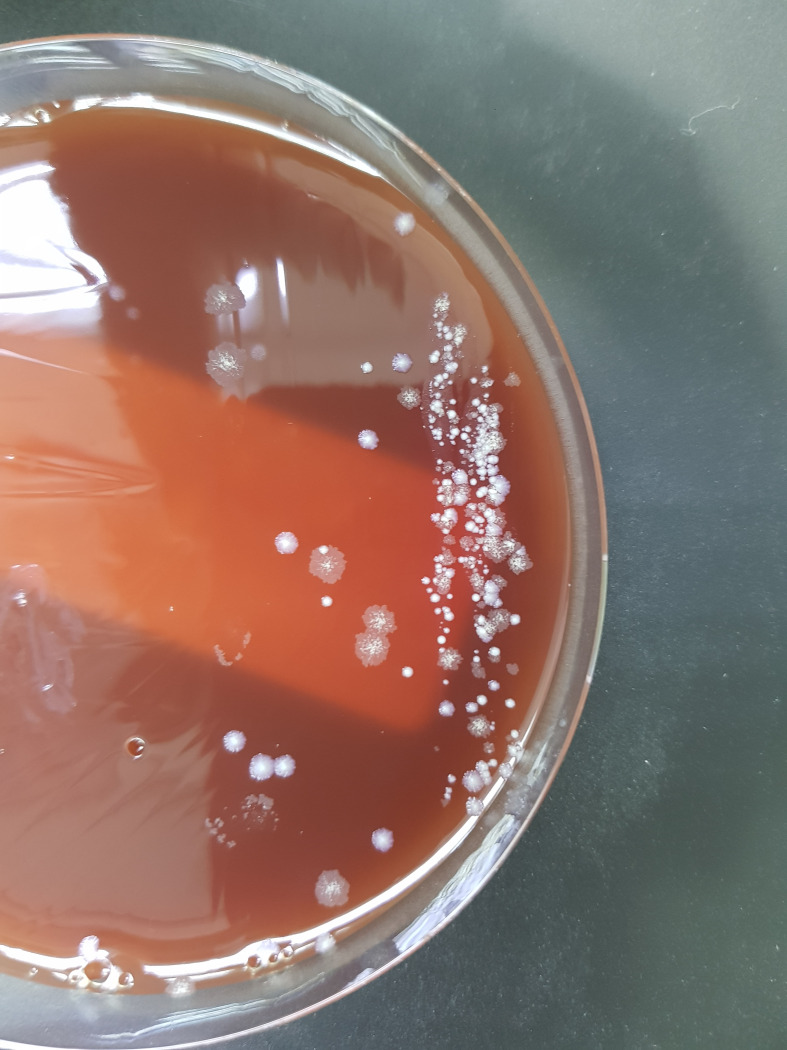
*
Mycobacterium abscessus* colonies on blood agar 5 days after incubation.

After the excision biopsy of the skin lesions (Fig. 2), samples were sent to the laboratory. The histopathological report was of a suppurative granulomatous infiltrate, with a necrotic and abscessed centre. The Ziehl-Neelsen staining was negative for BAAR, and the PAS-Hematoxylin staining negative for fungal elements, giving the diagnosis of granulomatous hypodermitis with probable infectious aetiology (Fig. 3). The microbiological study showed growth of atypical mycobacteria on chocolate and blood agar culture medium.(Fig. 4)

The sample was sent to a molecular biology laboratory to determine the specific atypical mycobacteria. Talenti protocol was followed, using the polymerase chain reaction (PCR) of the gene encoding for the 65 kDa protein. The restriction enzymes used were BstEII and HaeIII. Thus, it was possible to identify the atypical mycobacterium as *‘Mycobacterium abscessus’*.

Treatment consisted of surgical debridement of all lesions, accompanied by antibiotic monotherapy with azithromycin 500 mg daily for 6 months.

## Discussion

Atypical mycobacteria are acid-alcohol resistant, and aerobic bacilli [11]. This resistance to disinfectants can cause post-surgical and post-procedural infections. Although *MABSC* most commonly causes skin and soft tissue infections, it can also cause infections in almost all human organs. Infection can occur after inhalation, inoculation, ingestion or self-application of material contaminated by mycobacteria, transmission between people being exceptional [11].

Infections with these microorganisms frequently occur after surgical procedures, penetrating accidental trauma, intramuscular injections, and superficial abrasions [12]. Several cases of infections caused by atypical mycobacteria after tattoos [13], cosmetic treatments [14–16], botulinum toxin injections [17], implant surgeries [18], etc., have been described. Also, outbreaks of atypical mycobacterial infections related to contaminated instruments have been described in hospitals [19].

The increase of immunosuppressed patients may also be one of the reasons for the rise in mycobacterial infections. Also, in recent years there has been an increase in the demand for cosmetic procedures. The International Society for Aesthetic Plastic Surgery [20] in 2017 published a study, revealing the annual global aesthetic situation, showing an overall 5 % increase in cosmetic surgery interventions over a twelve-month span.

Minimally invasive aesthetic procedures, such as botulinum toxin application, acupuncture, mesotherapy, hormonal injections, etc., must be performed by trained professionals and in centres authorized for this purpose. Otherwise, they can cause serious health complications. Rivera-Olivero *et al*. [21] studied 49 patients undergoing mesotherapy procedures. Of these, 81.6 % had atypical mycobacterial infections, reflecting an alarming percentage.

In Paraguay, the direction of health, related and technology establishments, under the Ministry of Public Health, regulates the setting up of clinics, medical offices, and includes gyms [22]. There are several health problems in Paraguay. The sanitary regulations have not been updated as new procedures and technological advances have emerged. Furthermore, the absence of a comprehensive health law and the absence of professional fees do not allow the self-regulation of professions [7, 22, 23].

This lack of delimitation and regulation on the scope of mainly non-invasive aesthetic medical procedures, as well as the lack of professional supervision accompanied by the ease of access to drugs, leads to the performance of exclusively medical aesthetic procedures by people without this training. This situation can trigger a series of complications such as foreseeable infections and even death.

Conventional diagnostic techniques for detection of atypical mycobacteria are useful, however, their specificity and sensitivity are unsatisfactory [24]. Advances in molecular biology and diagnostic methods allowed the analysis of restriction fragment length polymorphism by the hsp65 gene [24, 25]. This gene encodes the 65 kDa protein present in all mycobacteria, whose restriction gives rise to different fragments characteristic of each species of *mycobacterium* [25]. Thus, by PCR techniques, the differentiation of each species can be carried out.

Identification of species among mycobacteria is valuable as it provides useful information on patient management and appropriate treatment [24]. Currently there are antimicrobial drugs of choice for the treatment of atypical mycobacteria. However, more research is needed to determine new treatment options as well as quick and inexpensive diagnostic methods for effective treatment.

In addition, to avoid similar problems in the future, Paraguay will have to restructure the health system, prioritizing and strengthening the health control of private clinics.

## References

[1] Lee M-R, Sheng W-H, Hung C-C, Yu C-J, Lee L-N (2015). *Mycobacterium abscessus* complex infections in humans. Emerg Infect Dis.

[2] Maureen Cassidy P, Hedberg K, Saulson A, McNelly E, Winthrop KL (2009). Nontuberculous mycobacterial disease prevalence and risk factors: a changing epidemiology, clinical infectious disMycobacterial Disease Prevalence and Risk Factors: A Changing Epidemiology, Clinical Infectious Diseases. Clin Infect Dis.

[3] Park SC, Kang MJ, Han CH (2019). Prevalence, incidence, and mortality of nontuberculous mycobacterial infection in Korea: a nationwide population-based study. BMC Pulm Med.

[4] Benwill JL, Wallace RJ (2014). Mycobacterium abscessus: Challenges in diagnosis and treatment. Curr Opin Infect Dis.

[5] Jones RS, Shier KL, Master RN, Bao JR, Clark RB (2019). Current significance of the *Mycobacterium chelonae*-abscessus group. Diagn Microbiol Infect Dis.

[6] Preece CL, Wichelhaus TA, Perry A, Jones AL, Cummings SP (2016). Evaluation of various culture media for detection of rapidly growing mycobacteria from patients with cystic fibrVarious Culture Media for Detection of Rapidly Growing Mycobacteria from Patients with Cystic Fibrosis. J Clin Microbiol.

[7] Frontanilla TS, Abente SG (2018). Reglamentación del ejercicio profesional en medicina y odontología en Paraguay: una necesidad de salud pública [REGULATION OF PROFESSIONAL PRACTICE IN MEDICINE AND DENTISTRY IN PARAGUAY: A PUBLIC HEALTH NEED] (in Spanish). Rev Bras Odontol Leg RBOL.

[8] Franco-Duarte R, Černáková L, Kadam S, Kaushik KS, Salehi B (2019). Advances in chemical and biological methods to identify microorganisms-from past to prChemical and Biological Methods to Identify Microorganisms-From Past to Present. Microorganisms.

[9] Esteban J, Navas E (2018). Tratamiento de las Infecciones producidas Por Micobacterias no tuberculosas [Treatment of infections caused by nontuberculous mycobacteria] (in Spanish). Enf Infeccios y Microb Clín.

[10] Bbosa G, Mwebaza N, Odda J, Kyegombe D, Ntale M (2014). Antibiotics/antibacterial drug use, their marketing and promotion during the post-antibiotic golden age and their role in emergence of bacterial resistance. Health.

[11] Sajduda A, Martin A, Portaels F, Palomino JC (2012). hsp65 PCR-restriction analysis (PRA) with capillary electrophoresis for species identification and differentiation of *Mycobacterium kansasii* and *Mycobacterium chelonae*–*Mycobacterium abscessus* group. Int J Infect Dis.

[12] Soilán B, Kawabata A, Salinas M, Paredes ME, Abente S (2008). Infección post quirúrgica por Micobacterias Atípicas de Crecimiento Rápido [Post-surgical infection by fast-growing atypical mycobacteria] (in Spanish). An Fac Cienc Méd.

[13] Kerkemeyer KL, Darby JD, Jack Green F (2020). Mycobacterium Abscessus infection of a new tattoo in an australian traveller returning from Bali. J Travel Med.

[14] Tung-Chen Y, Caballero-Cardona C (2017). Infección por micobacterias no tuberculosas tras cirugía estética [Mycobacterium fortuitum infection in plastic surgery. Success with 12 weeks clarithromycin and levofloxacin treatment] (in Spanish). Med clínica.

[15] Colás Orós CE, Lasso Olayo JM, Vallejo Germosen L (2015). Infección cutánea por micobacteria atípica [Atypical mycobacterial skin infection] (in Spanish). Emergencias.

[16] Schcolnik CA, Hernández CR, Vega MME, Arenas GR, Fernández MRF (2014). Lipotransferencia complicada con micobacteriosis atípicas [Complicated lipoblot with atypical mycobacteriosis. Report of two cases and review of the literature] (in Spanish). Reporte de dos casos y revisión de la literatura Gac Med Mex.

[17] Chen X, Jin Y, Torres KMT, Li B, Zhuo F (2019). *Mycobacterium abscessus* cutaneous infection secondary to botulinum toxin injection: A report of 2 cases. JAAD Case Rep.

[18] Rüegg E, Cheretakis A, Modarressi A, Harbarth S, Pittet-Cuénod B (2015). Multisite Infection with *Mycobacterium abscessus* after Replacement of Breast Implants and Gluteal Lipofilling. Case Rep Infect Dis.

[19] Desai AN, Hurtado RM (2018). Infections and outbreaks of nontuberculous mycobacteria in hospital settings. Curr Treat Options Infect Dis.

[20] International Society of Aesthetic Plastic Surgery (2018). El último estudio internacional muestra un incremento global en cirugía estética [The latest international study shows a global increase in cosmetic surgery] (in Spanish). https://www.isaps.org/wp-content/uploads/2018/11/2017-Global-Survey-Press-Release_SP.pdf.

[21] Alejandra Rivera-Olivero I, Guevara A, Escalona A, Oliver M, Pérez- Alfonzo R (2006). Infecciones en tejidos blandos por micobacterias no tuberculosas secundarias a mesoterapia. ¿Cuánto vale la belleza? [Soft tissue infections due to non-tuberculous mycobacteria following mesotherapy. What is the price of beauty?] (in Spanish). Enfermedades Infecciosas y Microbiología Clínica.

[22] Nacional G Ministerio de Salud pÚblica y Bienestar social. Dirección de Establecimientos de salud, Afines y Tecnología sanitaria. Requisitos para la habilitación de Gimnasios. http://vigisalud.gov.py/establecimientos/images/documentos/dependencias/establecimientos/Requisitos%20para%20habilitacion%20y%20registro%20de%20gimnasios.pdf.

[23] Borracci RA, Mauro VM (2015). El profesionalismo Médico, Los Modelos de Regulación y La Autonomía Profesional [Medical professionalism, regulatory models and professional autonomy] (in Spanish). rac.

[24] Bicmen C, Gunduz AT, Coskun M, Senol G, Cirak AK (2011). Molecular detection and identification of mycobacterium tuberculosis complex and four clinically important nontuberculous mycobacterial species in smear-negative clinical samples by the genotype mycobacteria direct test. J Clin Microbiol.

[25] Saifi M, Jabbarzadeh E, Bahrmand AR, Karimi A, Pourazar S (2013). HSP65-PRA identification of non-tuberculosis mycobacteria from 4892 samples suspicious for mycobacterial infections. Clinical Microbiology and Infection.

